# Glycosylation of HIV Env Impacts IgG Subtype Responses to Vaccination

**DOI:** 10.3390/v11020153

**Published:** 2019-02-13

**Authors:** Rebecca Heß, Michael Storcksdieck genannt Bonsmann, Dennis Lapuente, Andre Maaske, Carsten Kirschning, Jürgen Ruland, Bernd Lepenies, Drew Hannaman, Matthias Tenbusch, Klaus Überla

**Affiliations:** 1 Department of Molecular and Medical Virology, Ruhr-University Bochum, 44801 Bochum, Germany; rebecca.hess@rub.de (R.H.); michael.storcksdieckgenanntbonsmann@rub.de (M.S.g.B.); Dennis.Lapuente@uk-erlangen.de (D.L.); Andre.Maaske@ruhr-uni-bochum.de (A.M.); 2 Institute of Clinical and Molecular Virology, University Hospital Erlangen, Friedrich-Alexander University Erlangen-Nürnberg, Germany; Medical Immunology Campus Erlangen, FAU Erlangen-Nürnberg, 91054 Erlangen, Germany; klaus.ueberla@fau.de; 3 Institute of Medical Microbiology, University Hospital Essen, University Duisburg-Essen, 45122 Essen, Germany; Carsten.Kirschning@uk-essen.de; 4 Institut für Klinische Chemie und Pathobiochemie, Klinikum rechts der Isar, Technische Universität München, 81675 München, Germany; j.ruland@tum.de; 5 Immunology Unit & Research Center for Emerging Infections and Zoonoses, University of Veterinary Medicine (TiHo) Hannover, 30559 Hannover, Germany; Bernd.Lepenies@tiho-hannover.de; 6 Ichor Medical Systems, Inc., San Diego, CA 92121, USA; dhannaman@ichorms.com

**Keywords:** HIV env, glycosylation, antibody response, vaccination

## Abstract

The envelope protein (Env) is the only surface protein of the human immunodeficiency virus (HIV) and as such the exclusive target for protective antibody responses. Experimental evidences from mouse models suggest a modulating property of Env to steer antibody class switching towards the less effective antibody subclass IgG1 accompanied with strong TH2 helper responses. By simple physical linkage we were able to imprint this bias, exemplified by a low IgG2a/IgG1 ratio of antigen-specific antibodies, onto an unrelated antigen, namely the HIV capsid protein p24. Here, our results indicate the glycan moiety of Env as the responsible immune modulating activity. Firstly, in *Card9*^−/−^ mice lacking specific C-Type lectin responsiveness, DNA immunization significantly increased the IgG2a/IgG1 ratio for the Env-specific antibodies while the antibody response against the F-protein of the respiratory syncytial virus (RSV) serving as control antigen remained unchanged. Secondly, sequential shortening of the Env encoding sequence revealed the C2V3 domain as responsible for the strong IgG1 responses and TH2 cytokine production. Removing all potential N-glycosylation sites from the C2V3 domain by site-specific mutagenesis reversed the vaccine-induced immune response towards a Th1-dominated T-cell response and a balanced IgG2a/IgG1 ratio. Accordingly, the stretch of oligomannose glycans in the C2V3 domain of Env might mediate a specific uptake and/or signaling modus in antigen presenting cells by involving interaction with an as yet unknown C-type lectin receptor. Our results contribute to a deeper understanding of the impact of Env glycosylation on HIV antigen-specific immune responses, which will further support HIV vaccine development.

## 1. Introduction

Vaccines are among the most effective measures to prevent infectious diseases. The most common correlate of protection for licensed vaccines is a strong antibody response [1]. Unfortunately, the classical vaccination approaches using inactivated or live-attenuated viruses were either not successful or not applicable due to safety concerns in the context of an HIV vaccine. However, a comparison of the vaccine-induced immune responses from the large clinical HIV-1 vaccine trials suggests that the quality of the humoral immune response may be critically important for protection rather than merely its quantity. Particularly, the protection against HIV infection in the RV144 trial correlated with Env-specific IgG3 antibodies and the ability to induce ADCC [2]. In contrast, the same monomeric gp120 protein vaccine used in the Vax003 study induced higher overall antibody levels but could not provide protection [2,3,4,5]. Here, the dominating subtype has been IgG1, which seemed to be less potent in Fc-Receptor (FcR) mediated secondary functions as compared to IgG3 [6,7]. Based on their affinity for FcRs, murine IgG2a seems to be the functional analog of human IgG3 [8,9] and is strongly associated with antiviral Th1 immune responses in mice [10]. Intriguingly, mIgG2a has been shown to be superior to mIgG1 with regard to protection from viral infections [11,12]. Interestingly, concurrent immunization against HIV Env and Gag with either gene-based vaccines or virus-like particles resulted in a balanced IgG2a/IgG1 antibody response to Gag, but the Env-specific response in the same animal was strongly dominated by IgG1 [13]. The induction of IgG1 antibodies was also accompanied by a strong Env-specific TH2 response, as indicated by IL-5 and IL-13 producing CD4^+^+ T-cells. The bias towards IgG1 antibody responses to HIV Env was in line with the results from several other immunization/infection experiments [14,15,16]. Furthermore, differences in the T-helper cell response induced by Gag and Env were also observed in human volunteers which have been vaccinated with ALVAC-HIV, a canarypox vector encoding the HIV antigens [13]. Since both antigens were delivered by the same vector and route, we hypothesize that this bias may be attributable to an intrinsic property of HIV Env. The resulting antibody response might be insufficient to promote protection or viral clearance by FcR-mediated effector functions and thus might also provide a kind of immune escape for HIV. The differentiation of T-helper cell responses depends mainly on the interaction with the antigen-presenting cell (APC), in particular with the affinity of the TCR to MHCII/peptide complex, the expression of co-stimulatory molecules, and the cytokines produced by the APC [17,18]. The latter depend very much on the engagement of different pattern-recognition-receptors (PRR) [19,20,21]. Furthermore, the glycosylation pattern of an antigen has also been implicated in immune modulation by promoting certain antibody or T-cell responses [22,23,24,25]. Since, in contrast to Gag, HIV Env is heavily glycosylated, binding of Env to certain C-type lectin receptors (CLR) on different APC populations might influence T-cell priming and/or B-cell activation [26,27,28,29]. Some CLRs induce the activation of tyrosine kinases, such as Syk, through classical immunoreceptor tyrosine-based activation motifs (ITAMs) or hemITAMs. This leads to the assembly of a ternary protein complex consisting of the caspase recruitment domain-containing protein 9 (CARD9), B-cell lymphoma/leukemia 10 (Bcl10), and the mucosa-associated lymphoid tissue lymphoma translocation protein 1 (MALT-1). In contrast, other CLRs carry intracellular immunoreceptor tyrosine-based inhibitory motifs (ITIMs), thus they are capable of dampening cellular responses.

In this study, we evaluated these structural properties of HIV Env as potential drivers of the biased T-cell and/or antibody responses. We applied DNA vaccines encoding HIV Env or other viral surface glycoproteins, namely the F-protein from the respiratory syncytial virus (RSV-F) or hemagglutinin from influenza a virus (IAV-HA), to characterize the differential antibody and T-cell responses in mice. Furthermore, we addressed whether TLR- or CLR-signaling is important for the IgG1 bias by application of respective knock-out animals. Finally, we identified the glycosylation of HIV Env in the C2V3 region as critical modulator of the antibody response toward the less efficient IgG1 subtype.

## 2. Materials and Methods

### 2.1. Plasmids

**Figure 1:**
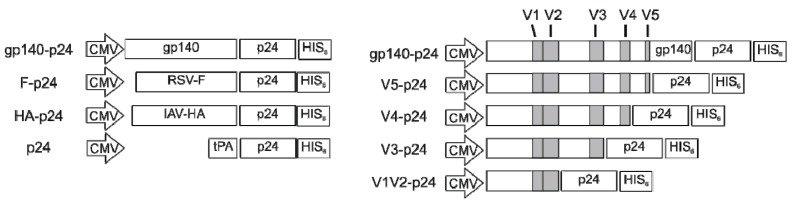
Maps of expression cassettes.

Initially, the expression plasmids Hgpsyn [30] and pConBgp140GC/D [31] carrying codon-optimized sequences for HIV Gag/pol or HIV Env, respectively, were used for DNA immunizations. Plasmids encoding the codon-optimized sequences of RSV-F or IAV-HA have been described previously [32]. For the direct comparison, new expression plasmids were generated based on the pVax vector and each encoded a fusion construct of the soluble ectodomain of the respective viral surface protein and HIV p24 as an internal control antigen. The ectodomains of HIV-Env, RSV-F, and IAV-HA were amplified without the transmembrane and intracytoplasmic domain by specific PCRs and cloned separated by a G4S linker at the N-terminal position of HIV p24 into pV-mDEC-HIVp24 [33]. The resulting plasmids are referred to as gp140-p24, F-p24 and HA-p24 respectively and lead to the expression of secreted fusion proteins. Since p24 alone is not secreted, an additional control plasmid, referred to as p24, was used in the immunization which mediates the secretion of a non-fused p24 by a TPA leader signal. Further Env–p24 fusion constructs were generated in which the gp140 was stepwise shortened by cutting after the respective variable region. All expression cassettes contain a CMV promotor and an additional HIS_6_-Tag at the N-terminus for purification purposes. A schematic overview on the ORFs of the new constructs is shown below in Figure 1.

### 2.2. Expression Analyses

To confirm antigen expression, cells of the human embryonic kidney cell line 293-T (HEK 293T; ATCC CRL-3216, LGC Standards, Wesel, Germany) were transfected with the respective plasmids. For 25-cm^2^ flasks, a formulation of 10 µg of plasmid DNA and 15 µg of polyethylenimine (PEI) in 500 µL serum-free DMEM (Life Technologies, Carlsbad, CA, USA) was used. Upon transfection, the cells were incubated in DMEM containing 1% FCS (Life Technologies) in a humid atmosphere with 5% CO_2_ at 37 °C and the supernatants were harvested 48 h post transfection. After clearance by centrifugation (940× *g* for 10 min), the cell free supernatants were mixed with SDS sample buffer and analyzed by Western Blot.

In brief, a SDS-PAGE was performed at a constant voltage of 140 V for 90 min followed by a transfer of the proteins on nitrocellulose membrane at 100 V for 1 h. Afterwards, blotted proteins were made visible by using antigen-specific antibodies for the respective encoded proteins (polyclonal a-F and a-HA antiserum from mice co-vaccinated with F and HA; polyclonal a-gp120, Acris, Herford, Germany; monoclonal a-p24, NIH AIDS reagent program) and matched secondary antibodies linked to horseradish peroxidase. Incubation with Chemi Glow West substrate solution (Protein Simple, San Jose, CA, USA) led to a chemiluminescent reaction which was recorded with a luminometer (Hamamatsu Photonics, Hamamatsu, Japan).

### 2.3. Mice and Immunization

All animal experiments performed during this study were approved by an external ethics committee authorized by the North Rhine-Westphalia Ministry for Environment and Nature Protection, Agriculture and Consumer Protection with the project licenses (AZ 84-02.04.2015.A082, approved at 28.07.2015). Six- to eight-week old BALB/cJRj and C57BL/6J mice were purchased from Janvier Laboratories (Le Genest-Saint-Isle, France) while *Myd88/Trif^−/−^* mice were provided by C. Kirschning [34]. *Card9^−/−^* mice were generated by Jürgen Ruland and backcrossed at the TiHo Hannover [35]. All mice were housed in individually ventilated cages in accordance to the national law and institutional guidelines at the animal facility of the Faculty of Medicine, Ruhr-University Bochum. DNA immunizations were performed as described previously [13]. Briefly, animals were anesthetized with ketamine/xylazine and received 15 µg of plasmid DNA in 30 µL of PBS in each hind leg followed by local application of an electric pulse (TriGrid; Ichor Medical, San Diego, CA, USA). A boost immunization following the same schedule was performed four weeks after the priming.

### 2.4. Determination of Humoral Immune Responses

Three and six weeks after the prime immunization, serum samples were collected and used to determine the humoral immune response by antigen-specific antibody-ELISAs. Ninety-six-well plates were coated with the respective antigen dissolved in 0.1 M bicarbonate buffer (pH 9.6) (RSV-F, Sino Biological, Beijing, China, 100 ng/well; IAV-HA, inactivated IAV-particles, 10^6^ PFU/well; gp140-his, own purification, 100 ng/well; Gag-GST, own purification, 150 ng/well) at 4 °C overnight. After washing with PBS-T, the coated plates were blocked with 5% skim milk powder in PBS-T and then incubated for 1 h at room temperature (RT) with the immune sera in a dilution range from 10^−3^–10^−4^ in 2% skim milk powder in PBS-T. Upon this, the plates were washed again and incubated for 1 h at RT with HRP-conjugated anti-IgG1 or anti-IgG2a antibodies (both BD Biosciences, Heidelberg, Germany) diluted in skim milk. The amount of bound antibody was measured by a luminescent reaction detected by a microplate luminometer (Orion, Berthold Detection Systems, Bad Wildbad, Germany). In order to allow a direct comparison, both detection Abs were verified to yield the same relative light unit values for a given amount of the respective IgG subclass antibodies.

### 2.5. Determination of Cellular Immune Response

To determine the cellular immune response, six weeks after the initial immunization the spleens were collected in 5 mL HBSS (Life Technologies) and single-cell suspensions were prepared using a 70 µm cell strainer. After red blood cell lysis, splenocytes were resuspended at a cell density of 10^7^/mL in RPMI 1640 (Life Technologies) supplemented with 10% FCS (Life Technologies), 1% penicillin/streptomycin (Life Technologies), 10 mM HEPES, 4 mM L-glutamine (Life Technologies), and 50 µM 2-mercaptoethanol. Intracellular cytokine staining was performed as previously described [13]. Briefly, 10^6^ splenocytes/well were seeded in a 96-well U bottom plate and stimulated with 5 µM of MHC-II restricted peptides (RSV-F: GWYTSVITIELSNIKE; IAV-HA: SFERFEIFPKE; HIV-Env: GVPVWKEATTTLFCASDAKA; HIV-Gag: PVGEIYKRWIILGLN and SPEVIPMFSALSEGA) in the presence of monensin (2 µM) and 1 µg/mL of an anti-CD28-antibody (BD Bioscience).

After 6 h incubation, the cell surface was stained with anti-mouse CD4-PerCP-eFluor710 and Fixable Viability Dye eFluor 750 (both eBioscience, Frankfurt a. M., Germany). The cells were then fixed with 2% paraformaldehyde and permeabilized using 0.5% saponin. For the detection of intracellular cytokines, anti-mouse TNF-α PE-Cy7, IFN-γ PE (both eBiosciece), and IL-2 APC (BD Bioscience) were used. Data were obtained with a FACS Canto II (BD Bioscience) and processed with FlowJo (Tree Star, Ashland, OR, USA).

For the cytokine-specific ELISA, 5 × 10^6^ cells/per well were seeded in 48-well plates and stimulated with the above-mentioned MHC II-restricted peptides (5 µg/mL). After two days of incubation, the culture supernatants were analyzed for the presence of the cytokines IL-4, IL-5, IL-10, and IL-13 by the cytokine specific Ready-set-go! ELISA (eBioscience) according to the manufacturer’s instructions. Optical densities were acquired with a Sunrise ELISA reader (Tecan, Crailsheim, Germany) at 450 nm with 620 nm as reference wavelength.

For the cytokine-specific ELISA, 5 × 10^6^ cells/per well were seeded in 48-well plates and stimulated with the above-mentioned MHC II-restricted peptides (5 µg/mL). After two days of incubation, the culture supernatants were analyzed for the presence of the cytokines IL-4, IL-5, IL-10, and IL-13 by the cytokine specific Ready-set-go! ELISA (eBioscience) according to the manufacturer’s instructions. Optical densities were acquired with a Sunrise ELISA reader (Tecan, Crailsheim, Germany) at 450 nm with 620 nm as reference wavelength.

### 2.6. Statistical Analysis

Two-tailed Mann-Whitney tests, two-tailed unpaired *t* tests, one-way ANOVA with Tukey’s post-test, Kruskal-Walli’s test with Dunn’s post-test signed rank test were performed as indicated in the figure legends using Prism 5.0 (GraphPad Software Inc., San Diego, CA, USA) 

## 3. Results

### 3.1. Differential Antibody Response to Viral Glycoprotein after DNA Immunization

**Figure 2:**
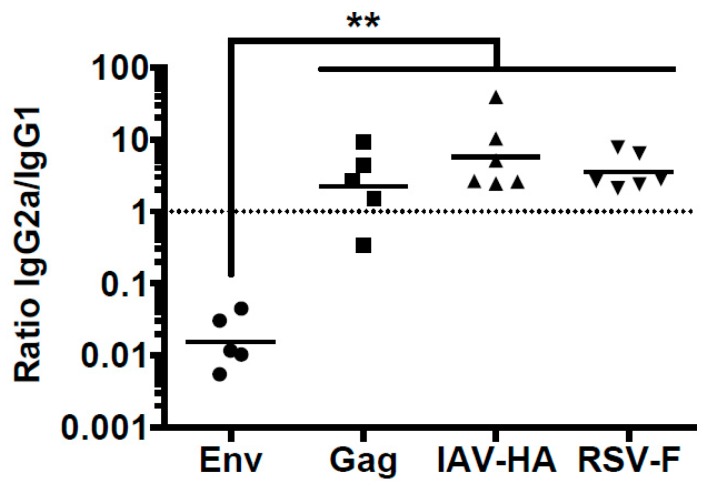
Differential humoral immune responses after DNA immunizations. Balb/c mice were immunized with either a combination of Env- and Gag-encoding plasmids (Hgpsyn and pConBgp140GC/D), or with RSV-F- or IAV-HA-encoding plasmids. Two weeks after the second immunization, antigen-specific IgG1 and IgG2a were determined via ELISA for each antigen separately and IgG2a/IgG1 ratios were calculated. Each Symbol represents an individual animal and geometric means are indicated by the solid lines. (*n* = 5–6, ** *p* < 0.01; Kruskal-Wallis with Dunn’s post-test).

Previously, we reported that the antibody response to HIV Env in mice differed from the one to HIV Gag even if the DNA vaccines encoding these two antigens were co-administered at the same site [13]. Since Gag and Env differ in a number of aspects, including their subcellular localization, we extended our comparisons by using other viral transmembrane proteins delivered by the same DNA vaccination protocol. Balb/c mice were immunized via DNA electroporation with plasmids encoding the HIV antigens, the F protein from RSV, or the HA from IAV. The analyses of the IgG subclass distribution for the antibody responses to Env, Gag, F, and HA revealed the excessive induction of IgG1 responses only for the Env antigen. Although F and HA were also expressed as membrane-anchored surface proteins, the antibodies targeting these proteins showed a balanced IgG2a/IgG1 ratio as observed before for the Gag-specific response (Figure 2). Therefore, we can exclude that expression of proteins on the surface of cells is sufficient for the induction of IgG1 dominated antibody responses.

**Figure 3:**
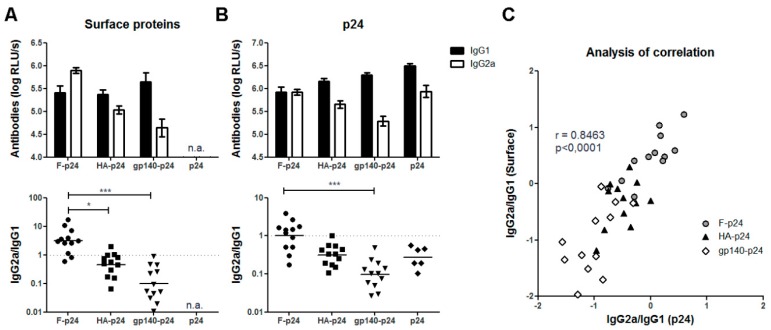
Antibody responses induced by DNA vaccines encoding p24 fusion proteins. Balb/c mice were immunized twice with the plasmids F-p24, HA-p24, gp140-p24, and p24, encoding fusions proteins as depicted in Figure 1. Two weeks after the booster immunization, the antibody levels specific for the respective surface proteins (**A**) or for the p24 antigen common to all fusion proteins (**B**) were determined by antigen-specific ELISAs (serum dilution 1/10,000). The RLUs for both IgG1 and IgG2a are depicted as mean values with SEM for the vaccine groups (top) and the ratio of RLUs for IgG2a to IgG1 were calculated for each mouse individually (bottom). The bars show the geometrical mean values (*n* = 6; * *p* < 0.05, ** *p* < 0.01, *** *p* < 0.001, Kruskal-Wallis-test with Dunn’s post-test). (**C**) Linear regression analysis (Pearson) between the IgG2a/IgG1-ratios to the surface protein and the fused p24.

This supports the notion that intrinsic properties of Env lead to the modulation of the antibody response. For an additional side-by-side comparison, expression plasmids were constructed which encode for fusion proteins of the soluble extracellular domain of the respective viral surface protein and the HIV p24, the capsid protein of HIV (Figure 1). Their application resulted in the expression and secretion of F-p24, HA-p24, and gp140-p24 fusion proteins, respectively. In this manner, differential budding or shedding from transmembrane proteins are circumvented and all proteins can be recognized by the immune systems in the same soluble, monomeric form. Furthermore, we included an additional antigen, the HIV p24, to control whether the viral glycoproteins also affect the immune response to the fused protein. After DNA immunization with these plasmids, the antibody responses to the glycoproteins as well as to p24 were characterized by ELISA. All antigens induced strong antibody responses and the overall levels were not substantially different. However, the response to RSV-F was dominated by antibodies of the IgG2a subclass whereas the Env-specific antibodies were again mainly of the IgG1 subtype (Figure 3A). The IgG2a/IgG1 ratio for the HA-specific antibodies was slightly below one in this experiment. Notably, the antibody responses to the fused p24 paralleled the IgG subtype distribution observed for the respective glycoprotein (Figure 3B). Specifically, antibodies to p24 in animals vaccinated with F-p24 were almost equally distributed to IgG1 and IgG2a whereas in gp140-p24 treated animals the ratios of IgG2a/IgG1 for p24-specific antibodies were significantly shifted to IgG1.

Immunization with an additional control plasmid encoding p24 secreted by a TPA leader sequence also induced a slightly higher IgG1 than IgG2a response. This is in contrast to our former results with full Gag and might be attributable to the different localization of Gag (intracellularly) and p24 (extracellularly). Given the strong correlation of the IgG2a/IgG1 ratios for F, HA, and gp140 with the respective ratio to p24 (Figure 3C), however, we conclude that the immune modulatory property of HIV Env also influenced the response to the fused p24.

### 3.2. HIV Env Modulates T-Helper Cell Response 

**Figure 4:**
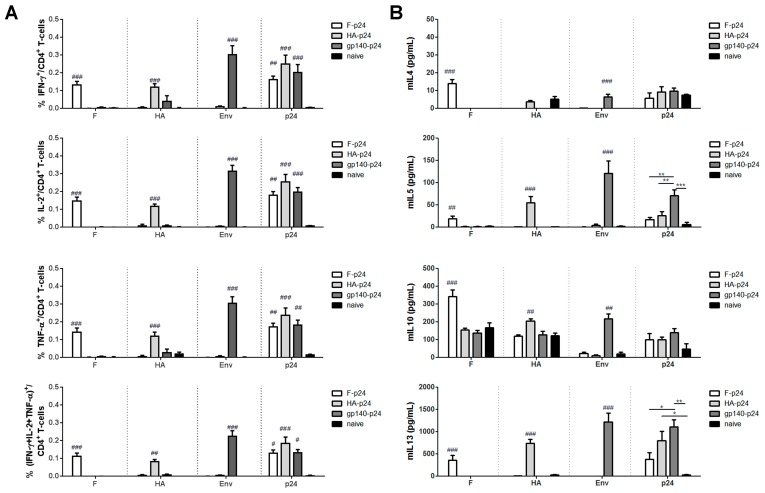
Cellular responses induced by DNA vaccines encoding p24 fusion proteins. Balb/c mice were immunized twice with DNA vaccines coding for the p24 fusion-proteins and antigen-specific CD4^+^ T-cell responses were analyzed two weeks after the booster immunization. Intracellular cytokine staining was used for the detection of the typical TH1 cytokines. The percentages of IFN-γ, TNF-α, and IL-2 producing CD4^+^ T-cells as well as polyfunctional triple positive cells are depicted in (**A**). The peptide used for the in vitro re-stimulation is shown at the x-axis of each graph. The analysis of TH2 cytokines was performed by cytokine specific ELISAs (**B**). In each graph, the bars represent the mean values with SEM of 4–6 mice (# *p* < 0.05 vs. naïve, ## *p* < 0.01 vs. naïve, ### *p* < 0.001 vs. naïve; * *p* < 0.05, ** *p* < 0.01, *** *p* < 0.001; one-way ANOVA with Tukey’s post-test).

The antigen-specific CD4^+^ T-cell responses were analyzed by intracellular cytokine staining and cytokine ELISA to explore whether the differential antibody responses might be a consequence of differences in the glycoprotein specific T helper cell responses. All vaccinated animals mounted specific T-cell responses to the respective glycoprotein as well as to the fused p24 as indicated by the capacity to produce typical TH1 cytokines, namely IFN-γ and TNF-α, after re-stimulation with cognate peptides (Figure 4A). Surprisingly, the percentages of cytokine-producing cells were even higher after stimulation with the Env peptide than after F or HA peptide stimulation. However, only the p24-specific response allows a direct comparison between the groups and this revealed no significant differences in TH1 responses induced by F-p24, HA-p24 or gp140-p24.

Since TH2 cytokines are more difficult to assess in the ICS, the levels of IL-4, IL-5, IL-10 and IL-13 were determined by ELISA in the supernatant after re-stimulation for 48 h (Figure 4B). High amounts of IL-5 and IL-13 were released by splenocytes from gp140-p24 immunized animals after stimulation with either Env or p24 peptides indicating the presence of Env- and p24-specific TH2 cells. In contrast, IL-4 and IL-10 were produced at comparable levels in all groups. Furthermore, cells from F-p24 treated animals produced significantly lower levels of IL-5 and IL-13 than the one from the gp140-p24 group. Taken together with the dominant IgG1 response, these results suggest that an intrinsic property of HIV Env leads to the induction of an unusual TH2 response possibly by modulating antigen-presenting cells.

### 3.3. CLR- but Not TLR-Mediated Signaling Might Be Involved in Env-Mediated Immune Modulation

**Figure 5:**
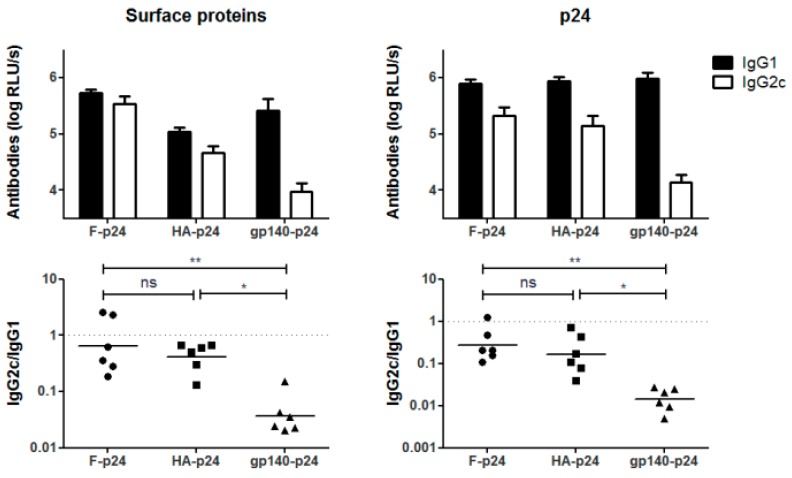
Biased immune responses to HIV Env in C57Bl6 mice. C57Bl/6J mice were immunized with the plasmids F-p24, HA-p24 and gp140-p24. The levels of IgG1 and IgG2c specific for the respective surface antigen (left) or the internal p24 (right) were determined by ELISA (top) and the IgG2c/IgG1 ratios were calculated for each individual animal represented by the symbols (bottom). The bars represent the mean values with SEM (top), whereas the lines represent the geometrical mean for each vaccine group (bottom). (*n* = 6; * *p* < 0.05, ** *p* < 0.01, ns = not significant, Kruskal-Wallis-test with Dunn’s post-test).

To address the question whether Env could modulate APCs directly by binding to and/or activation of different PRRs than the other two viral glycoproteins, we employed *Myd88/Trif^−/−^* and *Card9^−/−^* mice lacking TLR/IL-1 receptor and CLR functions, respectively. Since these knock-out strains were on the genetic C57Bl/6 background, we first demonstrated that the IgG1 bias for the Env-specific antibodies was not specific for Balb/c mice (Figure 5). Again, balanced IgG2c/IgG1 responses were measured for RSV-F whereas gp140-p24 mainly induced Env-specific IgG1 antibodies resulting in significantly different IgG2c/IgG1 ratios for these two groups. Importantly, the p24 specific response was modulated as well (Figure 5). The F-p24 and gp140-p24 immunogens were selected for a direct comparison, since these two immunogens showed the largest difference in wild type mice. Of note, similar levels of the TH2 cytokines IL-4, IL-5 and IL-13 were detected in supernatants of env-stimulated splenocytes ruling out possible differences in the cytokine levels due to the genetic background. 

**Figure 6:**
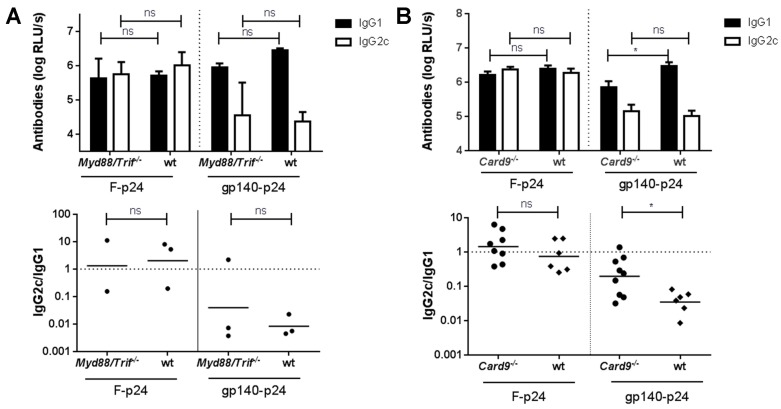
Impact of TLR- or CLR-signaling on the antibody response. *Myd88/Trif^−/−^* (**A**) or *Card9^−/−^* (**B**) mice were immunized with either F-p24 or gp140-p24 as described above and compared to WT C57Bl/6J mice. The levels of IgG1- and IgG2c specific for RSV-F or HIV-Env were determined by ELISA at a serum dilution of 1:1000 for both knock-out strains. Again, the IgG2c/IgG1 ratios were calculated for the individual animals represented by the symbols (*n* = 2–3 for *Myd88/Trif^−/−^*; *n* = 6–9 for *Card9^−/−^*). The bars represent the mean values with SEM (top), whereas the lines represent the geometrical mean for each vaccine group (bottom) (* *p* < 0.05, ns = not significant, Student’s *t*-test).

In *Myd88/Trif^−/−^* mice the humoral response to both antigens, F-p24 and gp140-p24, was not altered as compared to wild type controls (Figure 6A). Particularly, the F-specific response involved comparable levels of IgG1 and IgG2c whereas only low amounts of Env-specific IgG2c antibodies were detectable in both, the wild type as well as in the *Myd88/Trif^−/−^* animals. In contrast, a significant reduction of IgG1 antibodies was detected in *Card9^−/−^* mice immunized with gp140-p24 although IgG2c responses were unchanged in comparison to immunized wild type animals (Figure 6B). CARD9 is an essential adaptor molecule for several CLRs including Dectin-1, Dectin-2, Mincle, or Clec5a. Importantly, the reduction in IgG1 levels as compared to WT animals was not observed after vaccination with F-p24 (Figure 6B). These findings suggest that different glycosylation patterns of RSV-F and HIV Env and subsequent engagement of certain, not yet identified, CLRs may play an important role for the differential immune response.

### 3.4. Glycosylations in the C2V3 Region Determine the IgG1 Bias

**Figure 7:**
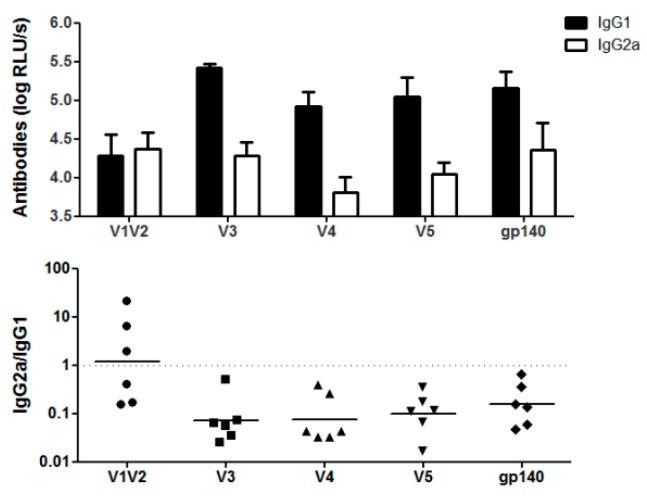
Humoral immune responses after vaccination with truncated variants of Env. Balb/c mice were vaccinated twice with the plasmids encoding the truncated Env-variants V1V2, V3, V4, V5, and the parental gp140 construct, depicted in Figure 1. Two weeks after the booster immunization, the Env-specific antibody responses were determined by ELISA. The upper part shows the levels of IgG1- and IgG2a-antibody response in each group (*n* = 6) as mean values with SEM. The lower part shows the ratio of IgG2a to IgG1 calculated individually for each animal with bars indicating the geometrical mean values. Each symbol represents an individual animal.

**Figure 8:**
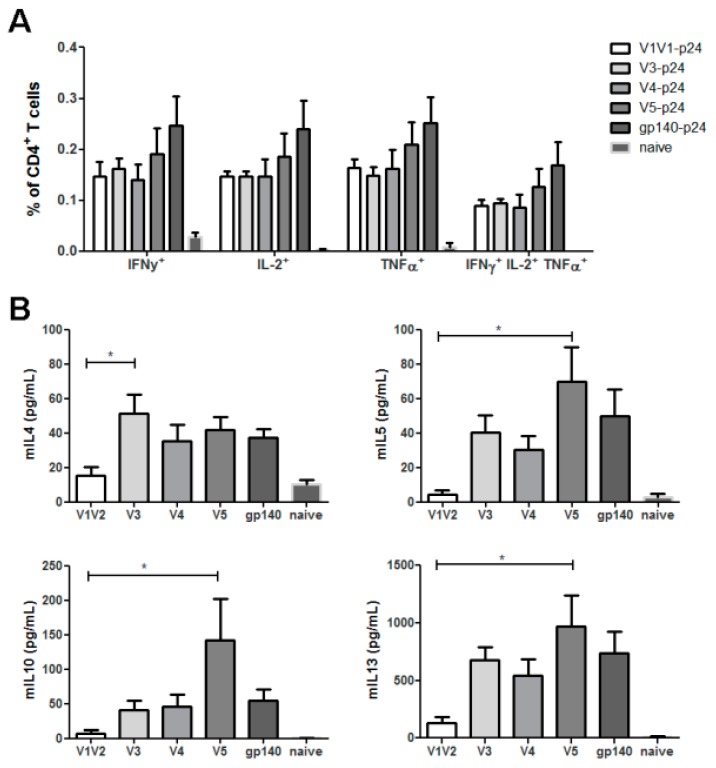
Cellular immune responses to the truncated variants of Env. Balb/c mice were vaccinated twice with the plasmids encoding the truncated Env-variants V1V2, V3, V4, V5, and the parental gp140 construct. Env-specific CD4^+^ T-cell responses were analyzed two weeks after the immunization. Intracellular cytokine staining was used for the detection of the typical TH1 cytokines and the percentages of IFN-γ, TNF-α and IL-2 producing CD4^+^ T-cells as well as polyfunctional triple positive cells are depicted in (**A**). In contrast, the analysis of TH2 cytokines was performed by a cytokine specific ELISA (**B**). In each graph, the bars represent the mean values with SEM of 4–6 mice (* *p* < 0.05, one-way ANOVA with Tukey’s post-test).

Next, we addressed the question whether certain domains of HIV Env are involved in the immune modulation by generating plasmids encoding p24 fusion proteins with partial deletions of the extracellular domain of gp140. Therefore, progressive C-terminal deletions of gp120 were fused to the p24 antigen as depicted in Figure 1. Immunization of Balb/c mice with the respective DNA constructs revealed the typical excess of Env-specific IgG1 antibodies in all groups except the one which received the shortest construct, namely V1V2-p24 (Figure 7). This group mounted IgG2a levels comparable to the other groups, but weaker IgG1 responses indicating a specific increase of Env-specific IgG1 by inclusion of the C2V3 domains. Interestingly, the antibody response to the V3-p24 protein was almost as strong as to the full-length gp140-p24 construct despite having fewer potential epitopes available due to its smaller size. To exclude a general impairment in the immunogenicity of V1V2-p24, we again analyzed the T-cell response against the immunodominant Env peptide which is present in all constructs. The ICS revealed no significant differences in the percentages of cytokine-producing CD4^+^ T-cells (TH1) in all vaccinated groups, although the responses in V5-p24 and gp140-p24 tended to be slightly higher (Figure 8A). Importantly, the CD4^+^ T-cell response in the group V1V2-p24 was similar to the remaining groups proving the immunogenicity of this construct.

**Figure 9:**
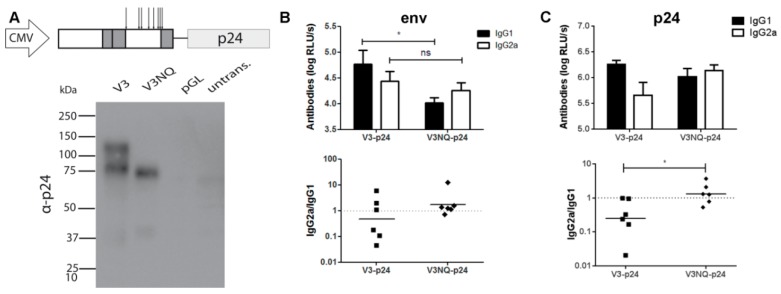
Impact of the glycosylation of the C2V3 region on the humoral immune response. (**A**) To delete all potential N-glycosylation sites from the C2V3 region, the Asn-coding triplets (indicated by arrows) in the construct V3-p24 were mutated to Gln-coding ones. 293T cells were transfected with the new construct, V3NQ, and deglycosylation of the Env variant was confirmed by Western Blot analyses of the supernatants with an p24-specific antibody. (V3 = glycosylated, V3NQ = not glycosylated, pGL = transfection control, untrans. = untransfected). Balb/c mice were immunized as described above with either V3-p24 or V3NQ-p24 and the antibody responses to Env (**B**) and p24 (**C**) were analyzed two weeks post boost immunization. The upper part shows the level of IgG1- and IgG2a-antibodies as mean values with SEM for six animals each (* *p* < 0.05, ns = not significant, Student’s *t*-test). The lower part depicts the ratio of IgG2a to IgG1 for each animal with bars indicating the geometrical mean values (* *p* < 0.05, ns = not significant, Mann-Whitney-test).

However, consistent with the reduced amount of IgG1 antibody response, Env-specific TH2 responses were nearly absent in this group as indicated by low amounts of IL-4, IL-5, IL-10, and IL-13. These cytokines became readily detectable when the C2V3 domains were present in the construct (Figure 8B). In accordance with the ICS data, the T-cell response to Env seemed to reach the maximum in the case of the V5-p24 construct. Since CLR signaling is involved in immune modulation, the glycosylation pattern of the C2V3 region might play an important role for the shift towards a TH2 response and subsequent IgG1 production. Therefore, we mutated all potential N-glycosylation sites to codons for glutamine in C2V3 resulting in the new construct, V3NQ-p24. The mutations led to the expected size reduction due to the missing glycosylation as revealed by Western Blot analyses of transfected 293 T-cells. However, both proteins were efficiently secreted into the supernatant and successfully fused to p24 (Figure 9A). Immunization with V3NQ-p24 induced IgG2a responses comparable to the fully glycosylated V3-p24, but the IgG1 response was significantly reduced (Figure 9B). Interestingly, the formerly imprinted bias in the antibody response to the fused p24 was also reverted and V3NQ-p24 immunized animals show a balanced IgG2a/IgG1 ratio (Figure 9C).

**Figure 10:**
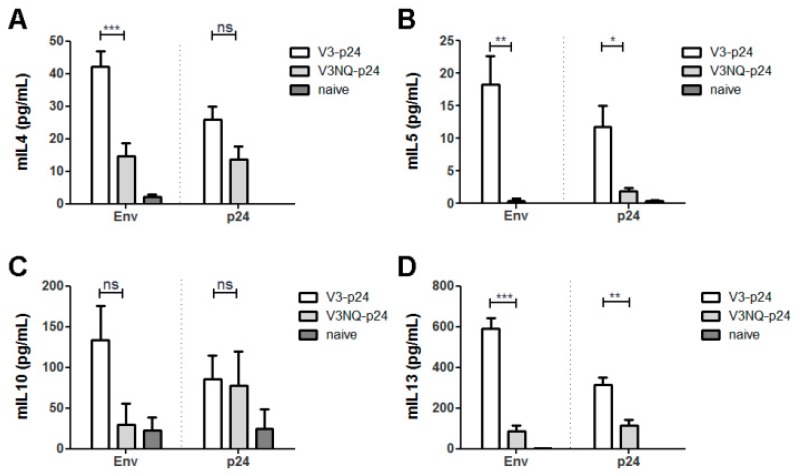
Impact of the glycosylation of the C2V3 region on TH2 responses. Balb/c mice were immunized twice in a 4-week interval with either V3-p24 or V3NQ-p24. Two weeks after the booster immunization, the cellular immune response to Env and p24 was analyzed via cytokine-specific ELISA. Depicted are the levels of the IL-4 (**A**), IL-5 (**B**), IL-10 (**C**) and IL-13 (**D**) as mean values with SEM (*n* = 4–6, * *p* < 0.05, ** *p* < 0.01, *** *p* < 0.001, ns = not significant, One-Way ANOVA with Tukey’s post-test).

This also confirmed the overall immunogenicity of the non-glycosylated protein. Finally, the TH2 responses were again addressed by cytokine ELISA. Here, the effect of the deglycosylation was even more pronounced with significantly higher levels of IL-4, IL-5 and IL-13 after Env restimulation in animals immunized with the fully glycosylated V3-p24. In contrast, splenocytes from V3NQ-p24 treated animals hardly produced IL-5 and IL-13 after stimulation with either Env or p24 supporting the hypothesis that the glycosylation pattern of Env directly impacts T-cell polarization (Figure 10).

## 4. Discussion

Several studies reported a predominant mouse IgG1 antibody response to the HIV Env protein after infection or vaccination which coincided with a strong induction of Env-specific TH2 T-helper cell responses [13,14,15,16]. Although substantial differences in the functional profile of human IgG1 and the murine counterpart exist, there are also several reports on dominating IgG1 responses to Env in HIV–infected individuals or gp120 vaccine recipients which do not seem to correlate with protection [3,36,37].

Since the bias in the antibody response to the unfavorable IgG1 subclass was observed for different vaccination approaches, including immunization using protein, DNA, or viral vector vaccines, an inherent property of the Env molecule seems to be responsible for this kind of immune modulation. In our previous study, the co-delivery of plasmids encoding either HIV Env or HIV Gag resulted in a balanced IgG1/IgG2a response towards HIV Gag, but in a TH2-dominated IgG1 response to HIV Env [13]. Since both antigens were delivered in the same muscle and T-cell priming most likely occurred in the same draining lymph node, this observation argues against a general Env induced cytokine milieu favoring TH2 responses. Supporting the idea of an inherent property of HIV Env, in this study, a DNA vaccine encoding HIV Env covalently linked to HIV p24 induced a biased antibody response not only to Env, but also to p24. This again was in sharp contrast to the other DNA vaccine encoding the p24 fused to RSV-F, which induced balanced IgG1/IgG2a responses to both antigens. By using the soluble fusion proteins, we also confirm that the biased antibody response is not a consequence of the membrane-anchored trimeric form of HIV Env. Since this bias was accompanied by the alteration of p24-specific CD4+ T-cell responses, a plausible explanation for the modulatory effect of HIV Env is the differential binding and/or signaling via PRRs on antigen-presenting cells. Although TLR-mediated immune activation was reported for HIV Env (TLR2/4) [38] as well as for RSV-F (TLR4) [39], immunization of *Myd88/Trif*^−/−^ mice did not reveal any impact on the vaccine-induced immune response excluding a TLR employment by Env. Although the small group sizes in this experiment might limit the significance, there were no changes in the overall antibody levels or the subtype distribution. In contrast, *Card9*^−/−^ mice lacking an essential adaptor protein for specific CLR signaling pathways displayed significantly reduced levels of Env-specific IgG1 antibodies, whereas the IgG2 response was not changed. Given the similarity of the IgG1 responses to RSV-F in wild type and *Card9*^−/−^ mice, our data exclude a general defect in the IgG1 antibody repertoire due to a lack of CARD9 function. Consequently, the extensive glycosylation of HIV Env might lead to the recognition by specific, not yet identified CLRs, which can directly impact APC maturation and subsequently T-cell differentiation. Potential candidate CLRs known for direct signaling via CARD9 are Dectin-1, Dectin-2 and Mincle which bind different glycan PAMPs (e.g., on mycobacterium tuberculosis or specific fungi) to support specifically TH1, TH2 or TH17 responses [22,23,24,40]. Since the bias in the antibody response was not completely absent from *Card9*^−/−^ mice, additional CLRs/PRRs might be involved in a cooperative immune modulation. Two additional receptors reported to interact with Env are the mannose receptor [28] and SIGLEC-1 on macrophages [41,42], the former of which mediates release of IL-10 [28] while the latter generally dampens innate and inflammatory immune activities [43,44].

Using plasmids encoding successively shortened Env variants, we were able to demonstrate that the presence of the C2V3 region led to the induction of Env-specific TH2 T-cell responses which coincided with a dominant IgG1 antibody response. Since the T-cells from all animals were stimulated with a single peptide located in the C1 region of HIV Env, differences in the T cell responses observed can not be due to differences in the epitope recognition. Thus, the results support the idea of a differential programming of the T-cells by APCs. Interestingly, the percentages of T-cells producing typical TH1 cytokines and the levels of IgG2a antibodies remained constant independent of the length of the protein. Since the results from the *Card9*^−/−^ mice supported the hypothesis of a crucial role for the glycan structure of HIV Env, we removed all eight potential N-glycosylation sites from the C2V3 region by N-to-Q mutation to revert the immune modulatory effect of this domain. Indeed, vaccination with the deglycosylated construct induced a balanced IgG2a/IgG1 antibody response and a significant reduction of TH2 cytokines compared to the fully glycosylated V3-p24 construct. Interestingly, nearly all glycan structures in the C2 region belong to the oligomannose class, whereas more complex glycans were reported to be found in V1/V2 [45]. The highly repetitive mannose structures are known ligands for a series of CLR, including the MR, DC-SIGN, Dectin-2 or Mincle. Furthermore, it has been reported that the amount of oligomannose residues on viral glycoproteins can have a direct impact on DC maturation. Recombinant HA produced in CHO cells in the presence of kifunensine had less complex glycan structures and higher numbers of high-mannose glycans which results in stronger upregulation of CD86 and CD40 in murine DC [46]. In accordance with the observation in *Card9*^−/−^ mice, binding of HIV Env via the mannose structures to receptors like Mincle or Dectin-2 might be also a possible explanation for differential signaling in APCs. Although all our experiments revealed a strong association of the TH2 T-cell response with the IgG1 predominance, we formally can not rule out partially T-cell independent effects on the B-cell response mediated by the glycosylation structure of HIV Env. It has been reported that HIV gp120 can bind and activate B-cells via DC-Sign and/or MR which finally result in class-switch recombination in B-cell when acting together with additional stimuli, like BAFF [47,48]. Since BAFF expression is induced by IL-4 and IL-10, it is also possible that the T-cell independent reaction is supported by the cytokine milieu. Although it is worthy to speculate that both T-cell dependent and independent mechanisms might contribute both to the biased antibody response, so far the T-cell independent activation of B-cells has not been yet linked to a certain subclass of IgGs.

Finally, with the results of the immunization study in the knock-out mice and the antibody responses to the truncated and deglycosylated Env variants we have two independent lines of evidence that the glycosylation of HIV Env is directly responsible for the reported immune modulation. Although we have not yet identified the CLRs recognizing HIV Env, we can speculate that either a particular subpopulation of APCs is targeted by the specific CLR/glycan interaction leading to the induction of specific TH2-polarized T-cells or the signaling within the APC is directly influenced by the binding, as it is reported for Mincle or Dectin-2, for example. It will be of great interest to identify the potential receptor and analyze in detail its role for this specific immune modulation, since inducing an unfavorable TH-2 dominated immune response may actually constitute an immune evasion mechanism. A deeper understanding of the influence of glycan structures on antigen-specific T-cell responses will be also of great value for the development of new vaccines targeting viral surface proteins.
